# Finite element analysis (FEA) of stress distribution in platform-switched short dental implants

**DOI:** 10.6026/973206300200248

**Published:** 2024-03-31

**Authors:** Khyati N Gosai, Vishwa Deepak Tripathi, Suman Yadav, Dhwani Vyas, PV Gopinath, Anuj Singh Parihar, Sajith Abraham

**Affiliations:** 1 Department of Prosthodontics Crown and Bridge, Maharaja Ganga Singh Dental College and Research Centre, Sri Ganganagar, Rajasthan, India; 2 Department of Periodontology, Bhabha Dental College, Bhopal, MP, India; 3 Maxillofacial and Dental Department, Numed Hospital, ITS Dental College, Muradnagar, India; 4 Department of Periodontology, KM Shah Dental College and Hospital, Sumandeep Vidyapeeth Deemed to be University, Piparia, Waghodia, Vadodara, Gujarat, India; 5 Department of Periodontics, Sree Anjaneya Institute of Dental Sciences, Modakkallur, Atholi, Kozhikode, Kerala, India; 6 Department of Periodontology, People's Dental Academy, Bhopal, Madhya Pradesh, India; 7 Department of Preventive Dental Sciences, College of Dentistry, King Faisal University, AlHassa, Saudi Arabia

**Keywords:** Finite element analysis, platform switch, short dental implants, stress

## Abstract

The distribution of stress on short platform switched dental implants is of interest. Hence, the mandibular posterior molar area was modelled using a three-dimensional finite element method (FEM) with a continuous 1.5 mm cortical bone thickness and an inner cancellous bone core. The implants used in the study were 5 mm long, 4.5 mm wide and 3.5 mm wide at the abutments. 120 N of force was applied in both the vertical and oblique (20° and 35°) directions to create a realistic simulation. ANSYS Workbench was generated for each model. Von Mises stress was assessed in the cortical and cancellous bones at varying depths. Ten noded tetrahedron elements with three degrees of freedom per node were employed to interpret translations on the x, y, and z axes. The stress-based biomechanical behaviour of platform switched short osseo-integrated implants varied across all 5 positions in FEM simulations, based on the depth of implant placement, the direction of applied force, and the shape of the bone. Data shows that opposite forces to the vertical forces caused more damage. Thus, the implantation of subcrestal implants resulted in reduced stress on the cortical and cancellous bone.

## Background:

Dental implants are frequently utilised to replace lost teeth [1]. Oral implants cannot be successful unless there is sufficient bone volume and density [2]. The way that pressures are absorbed by the surrounding bone has a major role in whether a dental implant succeeds or fails [3]. Depending on the implant's shape, the contact between it and the bone varies [2, 4]. These implants should be placed 1 to 2 mm below the bone crest [2]. Radiographic scans performed after a 5-year follow-up revealed that patients using the platform-switching approach had not displayed the resorption pattern. A dental implant is considered short if its length is less than 8 mm [5]. Numerous techniques, including strain gauges, photo-elastic models and finite element analysis (FEA), have been employed to examine the connection among loading, implant design, and peri-implant bone remodelling [2]. All of the structure's components have their stress and deflection properly computed [6, 7,8-9]. Finite element model (FEM) design provides information on stress and strain in bone and implants structures and facilitates the clear understanding of concepts for clinical applications involving any animals or humans [2]. Therefore, it is of interest to evaluate finite element analysis of stress distribution in platform-switched short dental implants.

## Materials and Methods:

This investigation was carried out at the department of oral implantology. The mandibular posterior molar region was modelled using a three-dimensional finite element method (FEM) with a consistently thick 1.5 mm cortical bone and an inner core of cancellous bone. The implants used in the study were 5 mm long, 4.5 mm wide and 3.5 mm wide at the abutments. 120 N of force was applied in both the axial and oblique (20° and 35°) directions to create a realistic simulation. Every model was created with Ansys Workbench. Von Mises stress was assessed in both cancellous and cortical bone at varying depths. To interpret translations on the x, y, and z axes, ten noded tetrahedron elements with three degrees of freedom per node were employed. The model was constructed using homogeneous, linearly elastic, and isotropic materials. Table 1 shows elastic features that have been reported in the literature. These investigations use fixed boundary conditions finite element modelling of the mandibular posterior area. The boundary condition is the use of power and control. The node on the muscle attachment where the boundary conditions were restricted was the external oblique line, which ran buccally to the lingual side of the mylohyoid ridge. The FEM assumed that the bone implant interface had an optimal fit between the implant and bone. Each model represents the loaded and osseo-integrated state.

## Results:

Cortical bone displayed higher stress in an oblique direction in the von Mises stress assessment for 0.5 mm subcrestal implants (35c). When cancellous bone is implanted subcrestally, low-stress values are seen. The lowest stress was recorded by implants positioned 1.5 mm subcrestally at 0 c. This was followed by an increase in stress oblique forces at 35 c. Similar to cortical bone, cancelous bone shows maximal stress in an oblique direction (35c) for subcrestal implants. The cortical bone displayed the largest stress concentration in an oblique orientation at 2 mm subcrestally, regardless of the force's angulation. At the equicrestal location, the cancellous bone had the greatest stress and the cortical bone experienced the least stress, regardless of the angulation of the load. Conversely, at the 1.5 mm subcrestal position in the subcreasatal position, the cancellous bone has the least stress, while the cortical bone experiences the most stress (Table 2).

## Discussion:

Crestal bone loss plays a significant role in deciding the implant's long-term prognosis. This can be avoided if the annual vertical bone loss surrounding an implant stays below 0.2 mm and does not surpass 2 mm in the first year. By doing this, the implant's biological breadth will be preserved [9]. Successful oral implants require adequate bone volume and density. The arch location is often a good indicator of bone quality [2]. Tomar *et al.* [9] evaluated the stress distribution around different thread design implants with and without platform switching in the maxillary posterior region. Load transmission mechanisms are influenced by platform switching, implant surface design and implantation site. Single thread design with platform switching is preferable because of reduced crestal resorption [9]. The impact of implant insertion depth on the distribution of stress in the bone surrounding dental implants that are Morse taper and platform-switched as shown by Ellendula *et al.* [2]. Hence, long-term success, platform-switched implants with Morse taper implantation sub-crestally (1-2 mm) is advised. Pellizzer *et al.* [10] assessed the impact of the platform-switching technique on stress distribution in implant, abutment, and peri-implant tissues using a 3-dimensional finite element analysis. The trabecular bone showed minimal stress that was evenly distributed [10]. Switching platform models produced maximum stress values that were lower and a factor of safety that was larger than one, both of which are regarded as acceptable values according to Menacho-Mendoza *et al.* [11]. Tapered implants raised the stress on the crestal bone when loaded as shown by Rasouli-Ghahroudi *et al.* [12]. Platform switching reduced the amount of stress that was transferred to the crestal bone in both tapered and parallel wall implants [12]. Further, the tapered implant shape actually raises the stress on the crestal bone [13]. According to Vijapure *et al.* [3], implants showed higher maximum main stress under oblique loading than under axial loading in every model. The maximum von Mises stress rose as the abutment's angulation increased [3]. The platform switching provides a simple and efficient way to manage the circumferential bone loss surrounding dental implants. The benefit of good reactions from both soft and hard tissue is another [12]. When compared to implants without platform switching, implants with platform switching placed less stress on the cortical bone surrounding the implant. Longer healing and improved tissue health are the outcomes of using a Morse taper implant system with platform switching, which improves communication between the implant and the intervening abutment [2]. In terms of the bone, all three models had the cortical bone around the implant's cervical location as the site of the largest von Mises stress [11]. Platform-switching implants offer an affordable, straightforward, and dependable biomechanical alternative [10]. It should be noted that the use of a single-piece implant to get over the difficulty of implant internal design modelling in two-piece implants is a bottleneck.

## Conclusion

Data shows that the maxillary posterior area is a suitable location for implant placement. Further, the cortical bone is under the maximum stress at the 0.5 mm subcrestal position for the cortical bone and the 1.5 mm subcrestal position for the cancellous bone, respectively.

## Figures and Tables

**Table 1:** Mechanical characteristics of titanium and bone utilised in this study

**Material**	**Young's modulus**	**Poisson's ratio**
Cancellous bone	1.15 GPa	0.41
Cortical bone	13.4 GPa	0.41
Titanium alloy	115000 (MPa)	0.28
Titanium	115.000 MPa	0.32

**Table 2:** The mean von Mises stress generated in the cortical and cancellous bone under a vertical and oblique load of 120 N

**Angulations of force**	**Cortical bone cancellous bone**					**Cortical bone cancellous bone**				
	Equicrest al	0.5 mm	1 mm	1.5 mm	2mm	Equicrest al	0.5 mm	1 mm	1.5 mm	2mm
0c	5.26	9.12	7.34	6.36	7.54	2.35	2.32	2.11	1.76	2.16
20c	9.75	19.31	17.37	17.14	18.23	3.14	2.35	2.42	2.32	2.67
35c	13.13	29.27	26.32	29.37	28.31	3.53	2.57	2.76	2.43	3.06
